# Soft tissue recurrent ameloblastomas also show some malignant features: A clinicopathological study of a 15-year database

**DOI:** 10.4317/medoral.20276

**Published:** 2015-02-07

**Authors:** Zitong Lin, Guowen Sun, Tiemei Wang, Qingang Hu, Fei Chen, Shanhui Wen

**Affiliations:** 1 Department of Dentomaxillofacial Radiology, Affiliated Stomatology Hospital of Medical School, Nanjing University, Zhong Yang Road 30, Nanjing, China; 2 Department of Oral and Maxillofacial Surgery, Affiliated Stomatology Hospital of Medical School, Nanjing University, Zhong Yang Road 30, Nanjing, China; 3 Department of Dentomaxillofacial Pathology, Affiliated Stomatology Hospital of Medical School, Nanjing University, Zhong Yang Road 30, Nanjing, China

## Abstract

**Background:**

To investigate the clinicopathological features of six cases of soft tissue recurrent ameloblastoma and explore the role of increased aggressive biological behavior in the recurrences and treatment of this type of ameloblastomas.

**Material and Methods:**

In this study, we retrospectively reviewed recurrent ameloblastomas during a 15-year period; six cases were diagnosed as soft tissue recurrent ameloblastoma. The clinical, radiographic, cytological and immunohistochemical records of these six cases were investigated and analyzed.

**Results:**

All the six soft tissue recurrent ameloblastomas occurred after radical bone resection, and were located in the adjacent soft tissues around the osteotomy regions. In Case 4, the patient developed pulmonary metastasis, extensive skull-base infiltration and cytological malignancy after multiple recurrences and malignant transformation was diagnosed. In the other five cases, although there were no cytological signs are sufficient to justify an ameloblastoma as malignant, some malignant features were observed. In Case 1, the tumor showed moderate atypical hyperplasia and the Ki-67 staining percentage was 40% positive, which are strongly suggestive of potential malignance. In Case 5, the patient developed a second soft tissue recurrence in the parapharyngeal region and later died of tumor-related complications. All the remaining three patients showed cytology atypia of varying degrees and high expression of PCNA or Ki-67, which confirmed active cell proliferation.

**Conclusions:**

Increased aggressiveness is an important factor of soft tissue recurrence. An intraoperative rapid pathological examination and more radical treatment are suggested for these cases.

**Key words:** Ameloblastoma, soft tissue recurrence, aggressive biological behaviour.

## Introduction

Ameloblastoma is the most common epithelial odontogenic tumor, and it accounts for 10% of all tumors that arise in the mandible and maxilla (1). Ameloblastoma is generally considered to be a benign lesion with locally invasive behavior and a tendency to reoccur after inadequate treatment (2-4). The risk of recurrence is reported to be significantly reduced by radical treatment compared to conservative surgical treatment (5,6). However, there are some reports of soft tissue recurrent ameloblastomas after radical treatment too. Olaitan retrospectively reviewed 26 cases of recurrent ameloblastoma within a 15-year period, and reported that four intraosseous ameloblastomas recurred in the soft tissues of the head and neck (3). Adebayo *et al*. described a case of soft issue recurrence in the chin 21 years after radical resection of an ameloblastoma of the mandible (7).

Recurrence ameloblastoma due to inadequate treatment is often emphasized in literatures; however, the mechanism of recurrence could be quite complex due to their highly variable biological behaviors. Ameloblastoma recurrence has been suggested to be correlated to both the type of surgical treatment and increased aggressive biological behavior (8). Ameloblastomas constitute a group of heterogeneous lesions that exhibit rather variable biological behaviors ranging from cystic expansion to aggressive solid mass formation and even malignant transformation (1). Furthermore, ameloblastomas occasionally demonstrate clinical course characteristic of malignancies and may be associated with significant morbidity and sometimes death (9). The World Health Organization (WHO) has classified ameloblastomas into the following variants: solid/multi cystic ameloblastoma, unicystic ameloblastoma, desmoplastic ameloblastoma and peripheral ameloblastoma, as well as malignant counterparts such as malignant ameloblastoma and ameloblastic carcinoma (10). Moreover, there are atypical ameloblastomas that showed cytology atypia but could not be classified as malignant ameloblastoma or ameloblastic carcinoma (11).

In this study, we retrospectively reviewed recurrent ameloblastomas during a 15-year period (1998-2013), six soft tissue recurrent ameloblastomas were diagnosed of 25 recurrent ameloblastomas. The soft tissue recurrent ameloblastomas which occurred after radical bone resection and located in the adjacent soft tissues and without involvement of the residual jaw, showed clincopathologcial features which were not quite consistent with intraossous recurrent ameloblastomas. The role of increased aggressive biological behavior in the recurrences and treatment are discussed in this paper.

## Material and Methods

We retrospectively reviewed the clinical data of ameloblastoma patients admitted to our hospital who underwent resection and had histopathologically confirmed recurrent ameloblastoma during 1998-2013. Of the 25 recurrent ameloblastomas, six cases were diagnosed as soft tissue recurrent ameloblastomas based on their clinical, radiographic and histological presentations. Of these six cases, three cases had multiple recurrences: Case 4 showed six recurrences during 1998-2013; cases 5, two recurrences during the period of study, and Case 6, had recurrences in 1992 and 1996 respectively. The following clinical data of the six recurrent cases were recorded for the six soft tissue recurrence cases: sex, age at presentation of recurrence, site of recurrence, time interval between primary operation and recurrence, number of recurrences, types of surgery and follow-up reviews.

Histopathological sections of the primary and recurrent lesions were collected and evaluated for the histopathologic type and presence of cytology atypia. The histopathologic classifications included follicular, plexiform, acanthomatous, granular, basal cell type, and keratoameloblastoma (4,12). The cytological atypic features included hyper cellularity, pleomorphism, hyper chromatic nuclei, and presence of mitotic figures (13).

Immunohistochemical examination for the quantitative expression of Ki-67 or semi-quantitative expression (-, + or ++) of antigen proliferating cell nuclear antigen (PCNA) was conducted in all the six recurrent cases (14,15).

All patients gave their written informed consent.

This study was approved by the Ethics Committee of the Affiliated Stomatology Hospital of Medical School, Nanjing University.

## Results

The clinical and pathological data of the six intraosseous ameloblastomas with soft tissue recurrence are shown in  Table 1.

**Table 1:**
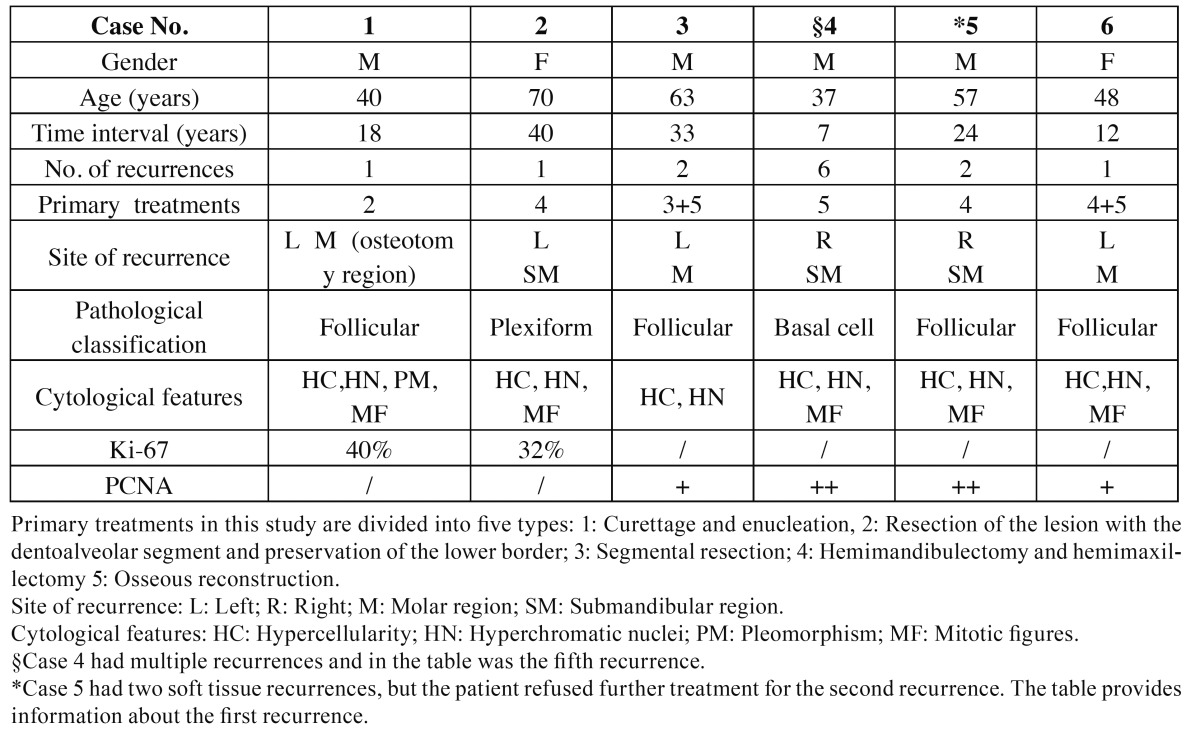
Clinical, pathological and immunohistochemical findings of six soft tissue recurrent ameloblastoma cases.

- Basal clinical data for recurrences

Four men and two women with soft tissue recurrent ameloblastoma were investigated in this study. The mean age at presentation of recurrence was 52.5 years (range, 37-70 years). The average time interval between the primary operation and recurrence was 22.3 years (range, 7-40 years). With the exception of Case 4, the time interval between the primary operation and the recurrences was ≥10 years in the remaining five cases.

- Type of primary surgery 

In Case 4, enucleation and curettage were performed for the primary maxillary ameloblastoma; further, radical bone resection (partial maxillectomy) was performed for the first recurrence 2 years later. In the other five cases, radical bone resection was performed for all the primary lesions: hemimandibulectomy in three cases, segmental resection in one case and resection of the lesion with the dentoalveolar segment and preservation of the lower border in one case. In two cases, osseous reconstruction was performed for the defects.

- Location of the recurrent tumors

In three cases, the recurrent tumors were located in the submandibular region on the same side of the primary lesion; in one case, it was located in the soft tissue of the osteotomy region; and in two cases, the tumors recurred in the gingival tissue of the molar regions.

- Follow-up review

In all six cases, extensive tumor excision was performed for the recurrent tumors to prevent further recurrences. The follow-up time was 2-10 years, and in four cases, there were no additional recurrences after that. However, in Case 4, the patient developed pulmonary metastasis and extensive skull-base infiltration after multiple recurrences and died in 2013; in Case 5, the patient developed a second soft tissue recurrence in the parapharyngeal region, but he refused further treatment and died not long after that due to complications caused by the tumor.

- Radiographic features

In all the six cases, all the primary lesions showed extensive bone destruction involving the cortical plate. With regard to the soft tissue recurrences, all the bone margins were totally intact and without signs of bone recurrence.

- Histopathological features

Of the six cases, four were of the follicular type; one was plexiform type and one, basal cell type. All the six patients presented with cytology atypia of varying degrees for both the primary lesions and recurrent lesions (13). In Case 4, the primary lesion showed hyper cellularity and the fifth recurrent tumor demonstrated increased cytological malignancy with hyper cellularity, hyper chromatic nuclei, cellular and nuclear pleomorphism, increased mitotic figures, and focal areas of necrosis. In the other five cases, there were no significant signs indicating malignant transformation (ameloblastic carcinoma ex-ameloblastoma). In Case 1 and Case 2, the percentage of Ki-67-positive cells was 40% and 32% respectively. The other four cases were positive for PCNA (+ in two cases, and ++ in two cases).

- Case Reports 

- Case 1

**Figure 1:**
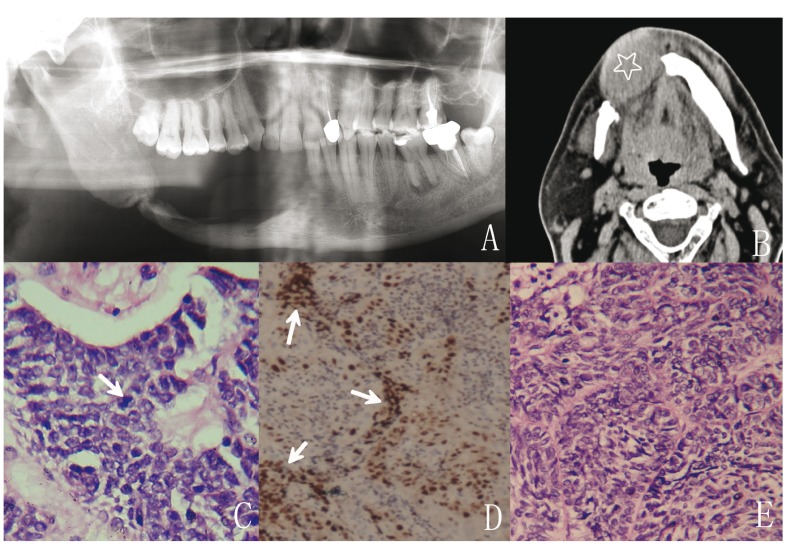
Radiographic, histological and immunohistochemical findings in Case 1. A: Panoramic radiograph showing a rectangular resection in the dentoalveolar side of the right mandible. B: Spiral CT image showing a soft tissue mass in the bone defect region (star). C: Pathological specimen of the recurrence showing moderate cellular atypia: hypercellularity, hyperchromatic nuclei, pleomorphism and mitotic figures (arrow). (hematoxylin and eosin staining; original magnification, ×400). D: Strong positive staining of the nuclei is seen in the tumor cells (Ki-67, arrow; ×100). E: Histopathological appearance of the primary lesion revealed hypercellularity, hyperchromatic nuclei and pleomorphism (hematoxylin and eosin staining; original magnification, ×200).

The patient was 22 years old at the time of the initial diagnosis of ameloblastoma in 1994. Resection of the lesion with dentoalveolar segment and preservation of the lower border was performed. Eighteen years later, the patient developed an intraoral swelling in the right mandibular body. CT revealed a soft tissue mass in the osteotomy region with an intact bone defect (Fig. 1A and 1B). Thereafter, extensive resection of the tumor and lower border of the jaw and transplantation of the ribs and fibula were performed. The final histological classification was recurrent follicular ameloblastoma. The patient has been followed up continuously with no evidence of recurrence. In the case of the recurrent tumor, moderate atypia was detected but it was not enough to justify an ameloblastic carcinoma. Moreover, the Ki-67 staining percentage was 40% which confirmed active cell proliferation (Fig. 1C and 1D). Further, retrospective evaluation of the histopathological sections of primary lesion also showed cytology atypia including hyper cellularity, hyper chromatic nuclei and pleomorphism (Fig. 1E).

- Case 2

The female patient was 30 years old at the time of the initial diagnosis of ameloblastoma, and hemimandibulectomy was performed in 1979. Thirty years later, the patient developed a swelling in the submandibular region. CT revealed a soft tissue mass with a totally intact bone defect. Extensive resection of the tumor was performed, and the histological diagnosis was recurrent ameloblastoma. The Ki-67 staining percentage was 32% positive for the recurrent ameloblastoma. Retrospective evaluation of the histopathological sections of both the primary and recurrent lesion showed plexiform ameloblastoma with cytology atypia including hyper cellularity, hyper chromatic nuclei and mitotic figures.

- Case 3

The patient was 30 years old at the time of the initial diagnosis of ameloblastoma in 1973. In 1992 and 2006, the patient had soft tissue recurrences located in the buccal mucosa and mandibular retromolar pad respectively. Extensive resections were performed for the recurrent tumors and the patient has been followed up with no evidence of recurrence. The histopathological classification was follicular ameloblastoma and cytology atypia was found for the primary lesions and two times of recurrent lesions.

- Case 4 

**Figure 2:**
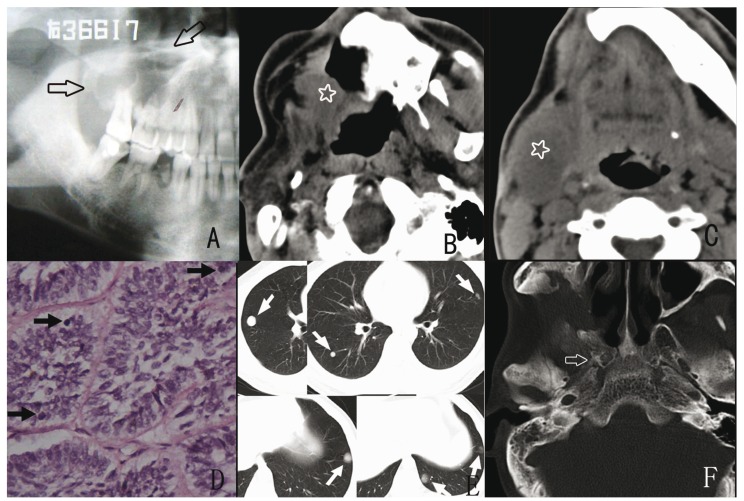
Radiographic and histological findings in Case 4. A: Panoramic radiograph from February 1999 showing a well-defined multilocular radiolucent shadow located from the second premolar to the maxillary tuberosity (arrow); B: Scan from 2006 showing an irregular soft-tissue density mass in the right pterygomaxillary fossa (star). C: Spiral CT images taken in 2007 showing soft tissue recurrences (star) behind the right submandibular gland. D: The specimen from 2007 depicting a region of cytology atypia with mitotic figures (arrows) and hyperchromatic nuclei (hematoxylin and eosin staining; original magnification, ×400). E: Thoracic CT images showing multiple nodules in both lung fields (arrows). F: Spiral CT images taken in 2008 showing erosion of the right petrosal apex of the temporal bone and the cranial base surface of the sphenoid bone with a diffuse boundary (arrow).

The patient was referred to our hospital and underwent total enucleation and curettage for maxillary ameloblastoma in 1999 (Fig. 2A). Histological examination showed a follicular ameloblastoma with slight cytology atypia. In 2001, the patient developed his first recurrence and a partial maxillectomy was performed. Following this, the patient had two more soft tissue recurrences in 2002 and 2004, and radical resection was performed on both occasions. All three recurrent tumors were treated at another hospital. In 2006, the patient was admitted to our hospital again with a soft tissue recurrence in the right pterygomaxillary fossa (Fig. 2B) and extensive tumor excision was performed. In 2007, the patient presented once again with recurrent ameloblastoma in the right submandibular region (Fig. 2C). And a thoracic CT examination indicated pulmonary metastasis (Fig. 2D). Extended tumor excision from the submandibular space was performed. Histological examination of the submandibular tumor specimen demonstrated cytological malignancy with hyper cellularity, hyper chromatic nuclei, cellular and nuclear pleomorphism, and increased mitotic figures (Fig. 2E). In 2008, the patient had the sixth recurrence that manifested as numbness of the right face. Craniofacial CT revealed infiltration of the skull-base, including the petrous bone (Fig. 2F). The patient did not undergo further treatment and died in 2013. Based on the entire aggressive clinic course and cytological progression, malignant transformation was diagnosed.

- Case 5

**Figure 3:**
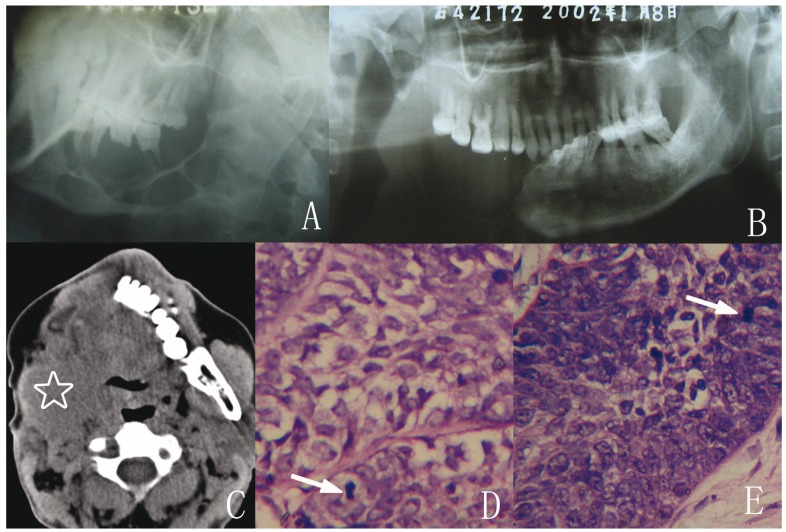
Radiographic and histological findings in Case 4. A: Lateral mandible radiograph from 1978 showing a multilocular radiolucent shadow spreading from the mandibular body to the ramus. B: Soft tissue recurrence in the submandibular region in 2002; the panoramic radiograph shows an intact bone margin of the mandible. C: Spiral CT scan from 2003 showing a second soft tissue recurrence in the right pterygomaxillary fossa (star). D and E: Histopathological appearance of the tumor in 1978 and 2002 depicting regions of cellular atypia with hypercellularity, hyperchromatic and nuclei mitotic figures (arrows) (hematoxylin and eosin staining; original magnification, ×400).

The patient was 33 years old at the time of the initial diagnosis in 1982 and hemimandibulectomy was performed for the primary lesion (Fig. 3A). Twenty-four years later, the patient developed a painful swelling in the right submandibular region with significant aggravation in a short time. Extra-oral examination revealed adhesion of a mass to the skin in the submandibular region. A panoramic radiograph revealed that the bone margin was completely intact (Fig. 3B). Extensive tumor resection was performed, and histological examination confirmed soft tissue recurrent ameloblastoma (follicular). Cytology atypia was confirmed for both the primary and recurrent lesions, based on the observed hyper cellularity, hyper chromatic nuclei and mitotic figures (Fig. 3C, 3D). One year later, the patient developed a second recurrence in the parapharyngeal region (Fig. 3E), but he refused further treatment and died not long after that.

- Case 6

The patient was 36 years old at the time of the initial diagnosis of ameloblastoma in 1989; hemimandibulectomy and osseous reconstruction were performed. Twelve years later, the patient developed a soft tissue recurrence in the mandibular third molar region. The final histological classification was recurrent follicular ameloblastoma. Similar with the other cases, retrospective cytological evaluation of the histopathological sections for the primary and recurrent lesion showed cytology atypia including hyper cellularity and hyper chromatic nuclei.

## Discussion

According to the present classification of ameloblastoma, the benign ameloblastomas are divided into 1) solid/multi cystic, 2) extra-osseous/peripheral, 3) desmoplastic, and 4) unicystic ameloblastoma. The solid/multicystic ameloblastomas are more aggressive than other types because they carry a high risk of local recurrences after inadequate treatment (1,4). Malignant ameloblastic lesions are divided into malignant (metastasizing) ameloblastoma and ameloblastic carcinoma. Malignant ameloblastoma describe an ameloblastoma with distant metastasis, and both primary and metastatic tumors retain a benign histological appearance. Ameloblastic carcinomas are tumors that combined morphologic features of ameloblastoma and carcinoma, regardless of the presence or absence of metastasis (13). However, ameloblastomas are on a complex spectrum of a single disease, there are atypical subtypes which presenting anaplastic features not sufficient to justify an ameloblastoma as malignant, but it carries increased aggressive biological behavior. In this study, some malignant features were observed for these cases at varied degrees. In case 4, pulmonary metastasis, extensive skull base infiltration and increased cellular atypia were found after multiple recurrences, and malignant transformation was diagnosed. However, in the other five cases, although there were no significant signs indicating ameloblastic carcinoma in both the primary and recurrent tumors, varying degrees of cellular atypia including hyper cellularity, hyper chromatic nuclei, pleomorphism, and mitotic figures were observed (13). High expression of Ki-67 or PCNA was also found, which confirmed active cell proliferation (14,15). In Case 1, moderate atypical hyperplasia and 40% positive staining for Ki-67 were observed, which is strongly suggestive of potential malignance (11,12,14). In Case 5, the patient developed a second recurrence in the parapharyngeal region and died of tumor-related complications. In all the remaining three cases, the tumors showed cytology atypia and high expression of PCNA or Ki-67, which confirmed active cell proliferation.

Ameloblastoma recurrence has been suggested to be correlated to both the histological features and the type of surgical treatment (8). It is known that ameloblastomas may recur from tumor tissue remaining in the residual stump of the jaw, from adjacent soft tissues, or from intraoperative contamination (16). However, in the present cases, tumors remaining in the stump of the jaw are less likely to recur because of the intact bone and recurrences occurring in the soft tissues around the osteotomy region. Moreover, although en-bloc bone resections had been performed before the soft tissue recurrences in all the cases, intraoperative contamination cannot be ignored; therefore, intraoperative contamination could have been a cause of tumor recurrence in the present cases. Nonetheless, it seems that residual tumor in adjacent soft tissue was the main cause of recurrence in the cases investigated in the present study. The soft tissue recurrent tumors in our study were aggressive, and the pre-surgical radiograph of the primary lesion showed destruction of the cortical plate and possible infiltration into the adjacent soft tissues. However, the high level of aggressiveness of the lesions was not noted at the time of surgery, and only radical bone resection was performed in all cases. Thus, from the present findings, it can be speculated that insufficient resection of adjacent soft tissue in the primary treatments and presence of residual tumor tissue in the adjacent soft tissues are the most likely causes of recurrence; this is in agreement with the observation of other published studies (17,18).

Considering the highly aggressive nature of these atypical ameloblastomas, firstly, a preoperative radiographic evaluation of whether the tumor involves the cortical plate is required, as destruction of the cortical plate suggests possible infiltration into the adjacent soft tissues. Secondly, a frozen rapid pathological examination during the surgery is suggested for these ameloblastomas cases; if cytological atypia indicative of aggressiveness is observed, more radical treatment including clearance of at least one more layer of tissue around such lesions should be performed (18). Moreover, for soft tissue recurrent ameloblastomas, a more radical treatment with enough safe margins is important to avoid secondary recurrences. Therefore, pathological examination of the resection edge is necessary for confirming the resection boundary in these cases.

It has also been reported that 50–80% of ameloblastoma recurrences are diagnosed within the first 5 years (2-4). However, in this study, late recurrence was found in five patients for whom the time interval between the primary operation and recurrence was ≥10 years; in agreement with this, late recurrences have been reported in some recent studies (7). Eckardt reported that the rate of cumulative relapses was 17% after 5 years and 19% after 10 years (6). Therefore, a long-term follow-up beyond 5 years is required, especially in cases where the tumors are of an aggressive nature.

## Conclusions

In this clinical retrospective study of recurrent ameloblastomas during a 15-year database (1998-2013), it was noted that soft tissue recurrence following radical bone resection also showed some malignant features. An intraoperative rapid pathological examination and more radical treatment are suggested for these cases of aggressive tumors.
